# Causes of mortality and pathological lesions observed post-mortem in red squirrels (Sciurus vulgaris) in Great Britain

**DOI:** 10.1186/1746-6148-9-229

**Published:** 2013-11-16

**Authors:** Victor R Simpson, Judith Hargreaves, Helen M Butler, Nicholas J Davison, David J Everest

**Affiliations:** 1 Wildlife Veterinary Investigation Centre, Chacewater, Truro, Cornwall TR4 8 PB, UK; 2 Abbey Veterinary Services, 89 Queen Street, Devon, Newton Abbot TQ12 2BG, UK; 3 PO Box 33, Ryde, Isle of Wight PO33 1BH, UK; 4 Animal Health and Veterinary Laboratories Agency Polwhele, Truro, Cornwall TR4 9AD, UK; 5 Bio Imaging Unit, Animal Health and Veterinary Laboratories Agency Weybridge, New Haw, Surrey, Addlestone KT15 3NB, UK; 6 Current address: Scottish Marine Animal Stranding Scheme, SAC Veterinary Services, Drummondhill, Stratherrick Road, Inverness IV2 4JZ, Scotland, UK

**Keywords:** Red squirrel, *Sciurus vulgaris*, Disease, Pathology, Neoplasm, Predation, Parasite, *Toxoplasma*

## Abstract

**Background:**

The red squirrel population in Great Britain has declined dramatically in recent decades, principally due to squirrelpox. Concern exists that red squirrels may become extinct nationally and, as there has been limited research in to diseases other than squirrelpox, this study aimed to identify additional causes of mortality.

**Results:**

Post-mortem examinations on 163 red squirrels found dead on Isle of Wight (IoW) England, in Scotland and at other locations in Great Britain showed that 41.7% (n = 68) were killed by road traffic and 9.2% (n = 15) by predators, principally domestic cats and dogs. The overall male/female ratio was 1.08/1. Fleas were recorded on 34.9% of IoW squirrels and on 43.8% of Scottish squirrels but sucking lice and ixodid ticks were only seen on Scottish squirrels. Bacterial infections were significant, particularly in association with respiratory disease (n = 16); two squirrels died of *Bordetella bronchiseptica* bronchopneumonia. Cases of fatal exudative dermatitis (n = 5) associated with a *luk*M-positive clone of *Staphylococcus aureus* occurred only on the IoW. Toxoplasmosis (n = 12) was also confined to IoW where it was responsible for almost one tenth (9.5%) of all deaths. Hepatozoonosis was common, especially in IoW squirrels, but was not considered a primary cause of mortality. Hepatic capillariasis affected four IoW squirrels and one from Scotland. Fungal infections included oral candidiasis, adiaspiromycosis and pulmonary phaeohyphomycosis. Neoplastic conditions diagnosed were: pulmonary carcinoma, gastric spindle cell tumour, renal papillary adenoma and trichoepithelioma. Epidermal hyperplasia of unknown aetiology was seen in squirrels showing crusty lesions of the ear pinnae on IoW (n = 3) and Brownsea Island (n = 1), associated in two cases with cutaneous wart-like growths. Miscellaneous diagnoses included chylothorax, electrocution, intussusception, suspected cholecalciferol rodenticide poisoning and foetal death and mummification. No cases of squirrelpox were diagnosed.

**Conclusions:**

Red squirrels in Britain suffer premature or unnatural mortality due to a number of conditions in addition to squirrelpox, many of which result, directly or indirectly, from human activities: road traffic trauma, pet predation, toxoplasmosis, trap injuries, rodenticide poisoning and electrocution accounted for 61% of all recorded mortality in this study. Red squirrels are also affected by several diseases of unknown aetiology which merit further research.

## Background

Historically, the red squirrel (*Sciurus vulgaris*) was a common and widespread species in Great Britain but the population has declined dramatically in recent decades. The principal reason for this is believed to be mortality caused by squirrelpox virus which is carried asymptomatically by the introduced American grey squirrel (*Sciurus carolinensis*) [1,2]. Grey squirrels have colonised most of mainland England and Wales and red squirrels are now largely confined to north west England and Scotland with relict populations persisting on several islands which have been kept free of grey squirrels. Despite concerns that red squirrels may become extinct in Britain there has been only limited research into causes of mortality other than squirrelpox and much of the published data relates only to specific conditions [3-7]. Keymer [8] in 1983 was the first to describe the range of diseases affecting red squirrels in Britain but there were few comparable studies until 2010 when LaRose et al. [9] conducted an extensive investigation in to causes of red squirrel mortality in Scotland. The purpose of the present study was, firstly, to investigate the causes of mortality and any associated pathological findings in red squirrels from geographically isolated populations in Britain, and secondly, to consider the factors that might influence the occurrence of disease in these populations. The results show that whilst trauma is the most commonly recorded cause of mortality, bacterial and parasitic infections are also important and that in some cases the presence or severity of infection varies markedly between geographic locations.

## Methods

During the period 2002–2012 post-mortem examinations were carried out on 163 red squirrels found dead or dying at various locations in the British Isles. Most had been found on or adjacent to roads or close to human habitations. They were judged to be juvenile, subadult or adult on the basis of their dentition, body dimensions and weight and on observer experience. A set of 112 squirrels from the Isle of Wight (IoW) were subjected to a basic post-mortem examination and tissue samples, which typically included heart, lung, liver, spleen and kidney, were fixed in 10% neutral buffered formalin. More detailed post-mortem examinations, which included microbiological examination where appropriate, were carried out on a further 14 squirrels from the IoW and on 32 from Scotland, three from Anglesey, Wales, and two from Cumbria, England. A formalin-fixed ear pinna was submitted from a squirrel found dead on Brownsea Island, England. Formalin-fixed tissue samples were embedded in paraffin wax, sectioned at 5 μm and stained by haematoxylin and eosin and, in selected cases, by special stains. Where gross lesions suggestive of a bacterial infection were seen tissue samples were inoculated onto 5 per cent sheep blood agar and MacConkey agar (Oxoid) and incubated aerobically at 37°C for 48 hr. Isolates were identified by their colonial morphology, Gram staining and their biochemical properties as determined using the appropriate analytical profile index (API-BioMerieux); suspect mycotic lesions were sent to the Mycology Reference Laboratory, Bristol. Lesions of suspected viral aetiology were submitted to Animal Health and Veterinary Laboratories Agency (AHVLA) Weybridge for examination by negative contrast stain transmission electron microscopy (TEM) [10]. Fresh spleen and faecal samples were screened for adenovirus infection by TEM and polymerase chain reaction analysis (PCR) [11]. Parasites were preserved in 70% ethanol or 10% formalin; fleas and ticks were submitted to the Royal Veterinary College and lice to the Natural History Museum for species identification. In selected cases formalin-fixed, wax-embedded tissue samples were submitted to Moredun Research Institute for examination by the immunoperoxidase test for *Toxoplasma gondii* antigen [12]. Statistical analysis was performed at AHVLA, Bury St Edmunds using Stata 12 (Stata corp, College Station, Texas). Proportions were compared pairwise between regions using Fisher’s exact test. Ratios were tested for parity assuming a binomial distribution. Kruskall Wallis test was used to test median weights between sexes. p = 0.05 was used for statistical significance. Weak evidence of a difference was regarded as 0.1 < p < 0.05.

## Results

Similar numbers of males (n = 84) and females (n = 78) were examined, a sex ratio of 1.08/1 (p = 0.695). The sex of one squirrel was not determined due to post-mortem damage. Many squirrels had suffered some degree of physical trauma and this, together with variation in the state of carcase preservation, meant that detailed gross and/or histopathological examination was not possible in every case.

Road traffic accidents were responsible for 41.7% (68 of 163) of mortality overall, with the figure for IoW (38.8%) similar to that for Scotland (43.8%). A diagnosis of road traffic death was based on a combination of case history and characteristic gross lesions. Male squirrels were possibly more likely to have died in road traffic accidents than females with a male:female incident ratio, adjusted for total male and female group sizes, of 1.41 (p = 0.080). Fifteen squirrels (9.2%) had puncture wounds, haemorrhage and fractures consistent with predation by domestic cats (n = 5), dogs (n = 6) and dogs or foxes (n = 4). Two squirrels on the IoW had suffered multiple limb amputations consistent with being caught in Fenn-type spring traps. Squirrel fatalities are summarised by geographic location, road traffic accidents and predation in Table 1.

**Table 1:** Numbers of squirrels examined per geographical region and the proportion killed by road traffic and by predators

	**IoW**	**Scotland**	**Anglesey**	**Cumbria**
Total carcases	126	32	3	2
Road traffic deaths	49 (38.8%)	14 (43.8%)	3 (100%)	2 (100%)
Predation	10 (7.9%)	5 (15.6%)	0 (0%)	0 (0%)

Comparisons between regions showed no significant differences.

### General condition

Most adult squirrels were in good body condition and the median body weights for adult males (306 g, n = 41) and females (313.5 g, n = 38) were not significantly different (p = 0.702). Overall, 32 squirrels were considered to be in poor or emaciated condition but only one of these was killed by road traffic, the rest dying of a range of infectious and non-infectious conditions. Infestation with ectoparasites was common and fleas (*Monopsyllus sciurorum*) were recorded in 34.9% (44 of 126) of squirrels from the IoW and in 43.8% (14 of 32) from Scotland (p = 0.413). However, although 34.4% (n = 11) of squirrels from Scotland were infested with sucking lice (mostly *Neohaematopinus sciuri* but also *Enderleinellus nitzschi*) and 25.0% (n = 8) infested with ticks (*Ixodes ricinus*) no lice or ticks were seen in squirrels from other locations (Table 2). In the absence of skin lesions carcases were not routinely examined for mites but *Dermacarus sciurinus* deutonymphs and *Metalistrophorus pagenstecheri* were seen in squirrels from IoW and *D. sciurinus* from Scotland. Larvae resembling *Neotrombicula autumnalis* were found on one squirrel from IoW. No ectoparasites were seen on the squirrels from Cumbria and Anglesey. Animals in poor or debilitated condition often had a higher than normal ectoparasite burden. Grossly evident anaemia was seen in two juvenile squirrels; one was heavily infested with lice and the other with fleas. However, in no case was the ectoparasite burden considered to be the primary cause of death, although it could have been a contributory factor.

**Table 2:** Principal ectoparasites found on red squirrels per geographical region

	**Number + ve/total examined (% + ve)**			
	**IoW**	**Scotland**	**Anglesey**	**Cumbria**
*Monopsyllus sciurorum*	44/126 (34.9)	14/32 (43.8)	0/3 (0)	0/2 (0)
*Neohaematopinus sciuri*, *Enderleinellus nitzschi*	0/126 (0^†^)	11^*^/32 (34.4^†^)	0/3 (0)	0/2 (0)
*Ixodes ricinus*	0/126 (0^†^)	8/32 (25.0^†^)	0/3 (0)	0/2 (0)

* *N. sciuri* n = 10, *E. nitzschi* n = 1.

^†^ Significant difference between IoW and Scotland (p < 0.001).

### Skin disease

**Figure 1:**
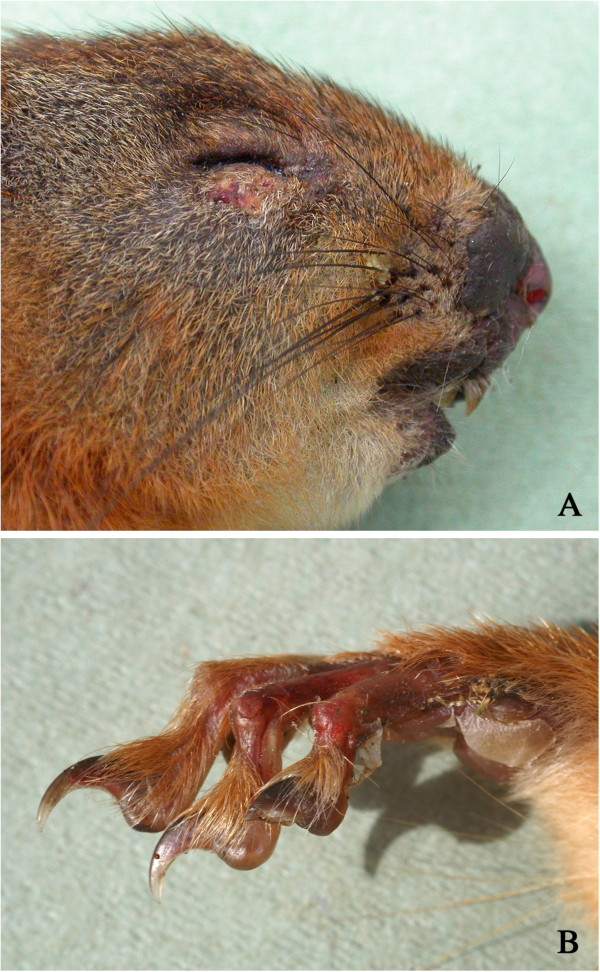
**Exudative dermatitis. A**: Scabby lesions around the mouth, nose and on the lower eyelid of a red squirrel that died of *Staphylococcus aureus*-associated exudative dermatitis. **B**: Sloughing of skin on the digits and the footpad. All four feet were affected.

The most important skin disease was a fatal exudative dermatitis associated with *Staphylococcus aureus* infection. Five squirrels were affected, all from the IoW, with exudative scabby lesions around the mouth and/or nose and, in some cases, the eyelids (Figure 1A). There was often inflammation and sloughing of the skin of the feet (Figure 1B), sometimes associated with ischemic necrosis of digits. Histologically there was an exudative, ulcerative, necrotic dermatitis with epidermal hyperplasia and hyperkeratosis. Numerous colonies of Gram-positive cocci were present both in the exudate and within intradermal pustules. Skin lesions were cultured from four squirrels and in each case *S. aureus* was isolated in pure or mixed culture. All the *S. aureus* isolates were of the same lineage and all possessed the leukotoxin M encoding gene (*luk*M). The lesions are described in more detail elsewhere [7,13].

**Figure 2:**
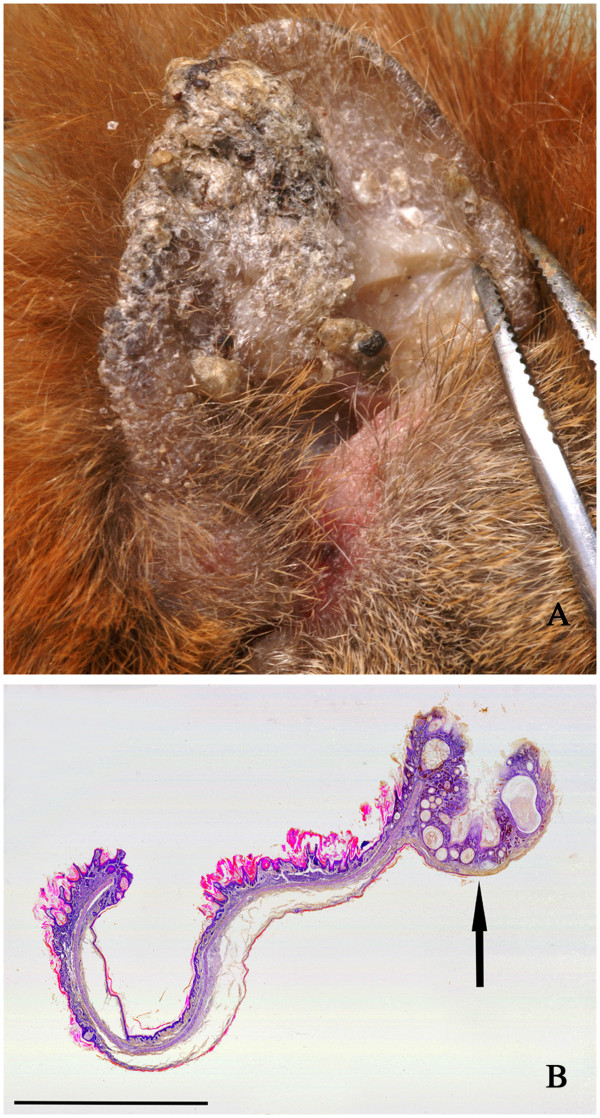
**Crusty thickening of the pinna. A**: Right pinna of a squirrel showing gross irregular thickening and keratinised crusts. The other ear was similarly affected. **B**: Histological section of a similar case to that seen in Figure 2A. There is epidermal hyperplasia, hyperkeratosis with rete ridges extending into the dermis and keratin-filled cysts. On the right margin there is also an ulcerated trichoepithelioma (arrow). Haematoxylin and eosin stain, bar = 5 mm.

**Figure 3:**
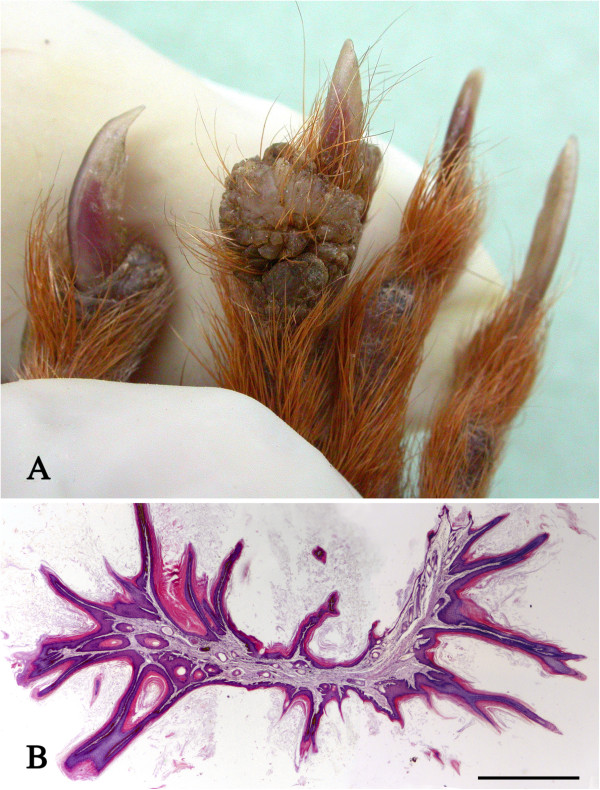
**Cutaneous wart–like lesions. A**: A proliferative wart-like lesion on a digit. Several similar lesions were present elsewhere on the same squirrel. **B**: Histological section of the lesion shown in Figure 3A showing keratinised papilliform proliferative projections of the epidermis. H & E stain, bar = 1 mm.

Four squirrels, three from the IoW and one from Brownsea Island had gross, crusty thickening of the pinnae, sometimes with keratinised or wart-like protuberances (Figure 2A). Histologically these lesions were associated with marked epidermal papilliform hyperplasia and orthokeratotic hyperkeratosis (Figure 2B). There was variable inflammatory cell infiltration of the dermis, no evidence of primary bacterial, fungal or parasite infections and no visible epidermal inclusions. However, in one case there was serocellular crusting and pustule formation and in a second case there was an ulcerated trichoepithelioma (Figure 2B). The squirrel in the latter case died of a pulmonary carcinoma but there was no apparent cause of death for the other two. No details were available for the squirrel from Brownsea with thickened ears but in two IoW cases wart-like skin lesions were also observed elsewhere, notably on the digits (Figure 3A). One such lesion examined histologically showed heavily keratinised papilliform proliferative projections of the epidermis but no significant inflammatory change and no evidence of an infectious agent (Figure 3B).

Other incidental skin lesions included a facial abscess from which *Yersinia enterocolitica* was isolated and a large ulcerated lesion over the scrotum associated with histological evidence of dermal necrosis and a coccal bacterial infection.

### Respiratory disease

Lungs from seven squirrels were examined bacteriologically and 142 histologically. Excluding those due to trauma, pulmonary lesions of varying degree were observed in 50 squirrels (35.2%). Protozoal schizonts identified morphologically as *Hepatozoon* sp. were frequently observed and the infection was commonest and heavier in squirrels from the IoW compared with those from Scotland (p = 0.014). Comparison with Cumbria was not possible due to the small number examined (n = 2) and no Anglesey cases were suitable for histological examination (Table 3). In most cases there was no significant inflammatory response associated with the schizonts and therefore their presence alone was not recorded as a lesion. However, in four heavily infected squirrels there was thickening of alveolar walls and infiltration by lymphocytes and macrophages. Heavy *Hepatozoon* infections were more common in squirrels suffering from other significant respiratory diseases.

**Table 3:** Principal endoparasites identified histologically in red squirrels per geographical region

	**Number + ve/total examined (% + ve)**			
	**IoW**	**Scotland**	**Anglesey**	**Cumbria**
*Hepatozoon* sp	38/116 (32.7^†^)	2/24 (8.3^†^)	u/s	0/2 (0)
*Toxoplasma gondii*	12/77 (15.6^*^)	0/24 (0^*^)	u/s	0/2 (0)
*Capillaria hepatica*	4/77 (5.2)	1/17 (5.9)	u/s	0/1 (0)

^†^ IoW and Scotland (p = 0.014).

^*^ IoW and Scotland (p = 0.064).

Specimens from Anglesey were unsuitable for examination (u/s).

**Figure 4:**
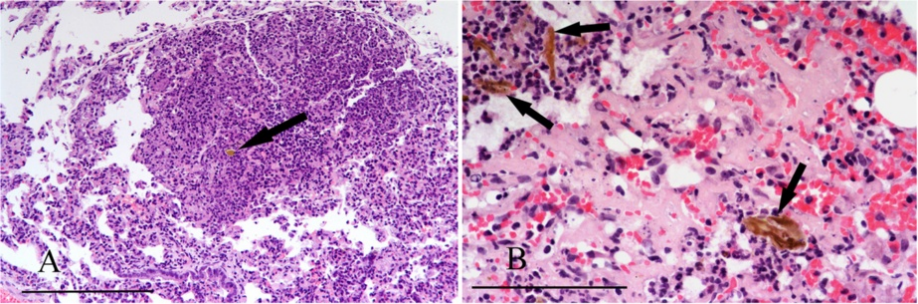
**Inhalation pneumonia. A**: Histological section of lung showing a mass of necrotic cell debris and neutrophils occluding the lumen of a bronchiole. Within the mass there is an amorphous foreign body, possibly inhaled ingesta (arrow). Numerous coccal bacteria were present within and around the lesion. H & E Stain, bar = 200 mμ. **B**: A second example of inhalation pneumonia shown at higher magnification with foreign bodies (arrows) surrounded by inflammatory cells and haemorrhage within the pulmonary parenchyma. H & E Stain, bar = 100 mμ.

Bacterial infections (n = 16) were important and included cases of inhalation pneumonia (n = 4), bronchopneumonia (n = 6) and focal parenchymal abscessation (n = 2). In cases of inhalation pneumonia the predominant lesions were in the apical lobes with focal inflammation and consolidation around the bronchi and/or bronchioles, necrosis and infiltration by neutrophils and macrophages. Within the lumen of the airway there was typically a mass of necrotic cell debris, neutrophils, bacteria and foreign material that appeared to be ingesta (Figure 4A); in some cases this foreign material extended into the parenchyma (Figure 4B). One of the affected squirrels also had oral lesions which may have predisposed to inhalation of foreign material and *Staphylococcus aureus* was isolated from both the oral lesion and the lung.

**Figure 5:**
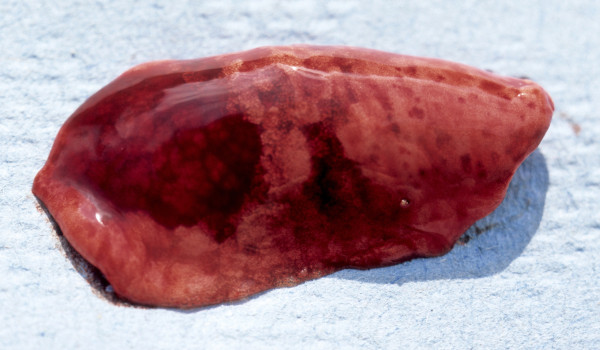
**Bordetella bronchopneumonia.** A *Bordetella bronchiseptica* infected lung showing multiple irregular foci of intense inflammation and consolidation.

**Figure 6:**
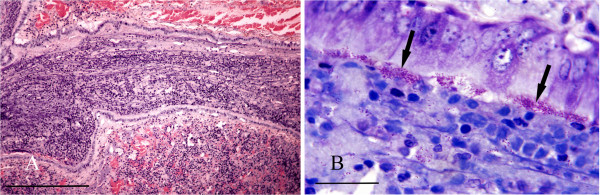
**Bordetella bronchopneumonia. A**: Histological section of lung showing a bronchiole occluded by a mass of inflammatory cells within a mucofibrinous matrix. H & E Stain, bar = 300 mμ. **B**: High power view of lung showing masses of *B. bronchiseptica* organisms (arrows) adhering to bronchiolar epithelium. Giemsa stain, bar = 25 mμ.

Bronchopneumonia due to *Bordetella bronchiseptica* infection was confirmed in a squirrel that was observed to die in respiratory distress. Gross examination of lungs showed large areas of intense inflammation and consolidation (Figure 5) from which *B. bronchiseptica* was isolated in pure culture. Histologically many bronchi and bronchioles were seen to be occluded by masses of degenerate inflammatory cells together with numerous Gram negative coccobacilli contained within a mucofibrinous matrix (Figure 6A). The surface of the lining epithelium was covered in an almost continuous layer of coccobacilli (Figure 6B), large numbers of which were also present together with mononuclear cells and proteinaceous fluid in the adjacent consolidated parenchyma. There was also a heavy *Hepatozoon* sp. infection. Almost identical lesions were observed in a second case that died concurrently in the same garden; similar lesions, although with heavy neutrophil infiltration, were seen in a third concurrent case at the same location. Neither of these latter cases was examined bacteriologically.

**Figure 7:**
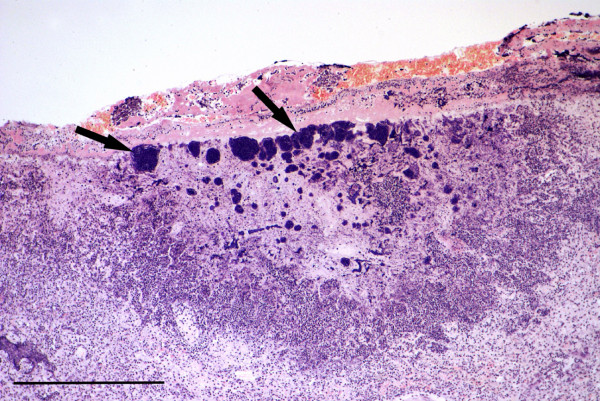
**Pulmonary abscess.** Lung with a subpleural abscess containing numerous colonies of Gram positive cocci (arrows) surrounded by inflammatory cells and collapsed parenchyma. H & E Stain, bar = 500 mμ.

A squirrel with congested, collapsed and partly autolysed lungs had intracytoplasmic inclusions in mononuclear cells resembling those of *Chlamydia* sp. Two squirrels died of pulmonary abscessation associated with bacterial infections. One was an adult female from Scotland which had a severe jaw abscess and focal inflammatory lesions in the pulmonary parenchyma with central necrosis and numerous bacilli present. The second was an adult female that died in thin condition on IoW and histological examination showed numerous colonies of Gram positive cocci in subpleural microabscesses surrounded by necrotic debris and a dense outer zone of neutrophils and macrophages (Figure 7). *Staphylococcus* sp infection was suspected but not confirmed as tissues were not submitted for bacteriology.

**Figure 8:**
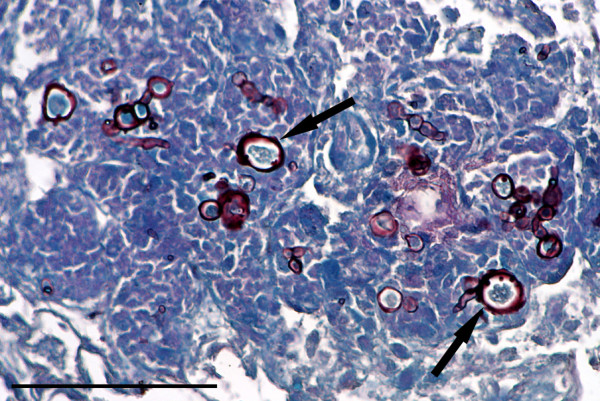
**Pulmonary phaeohyphomycosis.** Dark staining chlamydospores (arrows) are clearly visible in this granulomatous lung lesion, despite moderate autolysis. Grocott stain, bar = 25 mμ.

Phaeohyphomycosis was diagnosed in a squirrel that had suffered distal amputations and trauma to three limbs, probably due to it being caught in a spring trap. Histological examination showed granulomatous foci in the lungs containing groups of pigmented chlamydospores of variable size. Small hyphal structures extending from the spores were evident in sections stained by Grocott silver stain (Figure 8). The identity of the organism was not established as tissue was not cultured.

**Figure 9:**
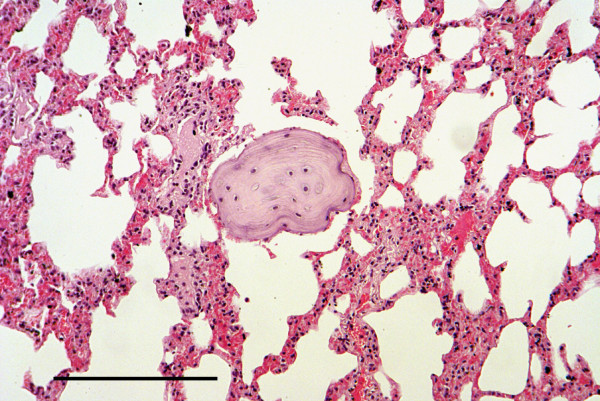
**Pulmonary metaplastic bone.** Section of lung showing a focus of metaplastic bone in the parenchyma. H & E Stain, bar = 200 mμ.

Fungal adiaspores typical of *Emmonsia crescens* were associated with a mild granulomatous reaction in the lungs of a squirrel that was found dead in emaciated condition. Four squirrels had foci of metaplastic bone or osteoid within the pulmonary parenchyma (Figure 9); two came from Cumbria, one from Scotland, and one, which also had pulmonary granulomata, from IoW.

**Figure 10:**
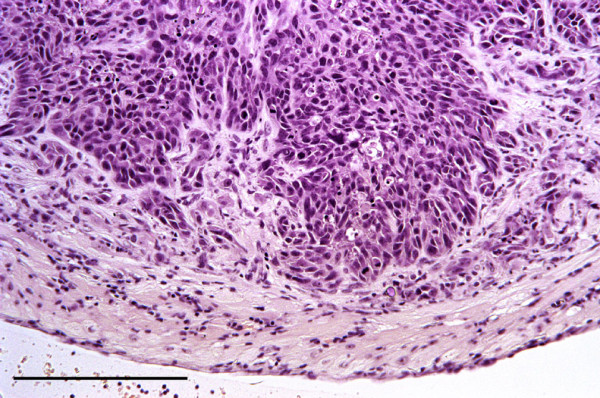
**Pulmonary carcinoma.** Carcinoma in lung composed mostly of polyhedral epithelial cells within a fibrovascular stroma. H & E Stain, bar = 200 mμ.

A pulmonary carcinoma caused the death of a subadult female squirrel on IoW. It was found dead in a garden in thin condition and with a heavy flea burden. Histological examination revealed an extensive invasive mass in one lung composed of innumerable islands of pleomorphic epithelial cells separated by a fibrovascular stroma (Figure 10). Enclosed within the mass were numerous alveoli, bronchial glands and foci of necrosis and inflammatory cells. The squirrel also had a trichoepithelioma affecting one pinna together with epidermal hyperplasia and hyperkeratosis.

**Figure 11:**
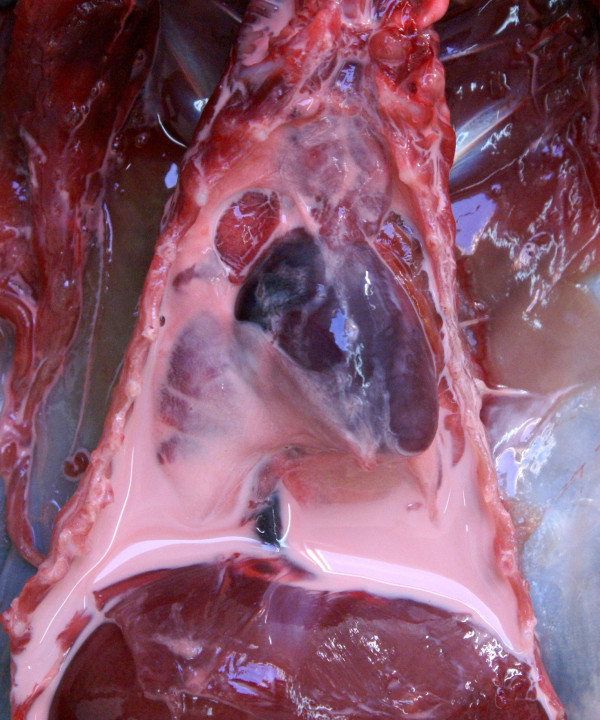
**A case of chylothorax.** The thoracic cavity is filled with pink stained chyle and the lungs are collapsed.

Chylothorax was diagnosed in a subadult squirrel from Scotland. The thoracic cavity was full of pinkish white, thick fluid and the lungs were collapsed apart from a few small localised areas (Figure 11). Both jugular veins were greatly distended. The squirrel had a purulent abscess circa 7 mm diameter in the left caudal pharynx and the lower incisor tooth on that side was markedly longer than that on the right. The thoracic fluid was confirmed to be chyle by direct microscopic examination, examination of Giemsa stained smears and by centrifugation. A light pure growth of *Hafnia alvei* was isolated from the fluid but the pharyngeal abscess produced a mixed growth of organisms.

**Figure 12:**
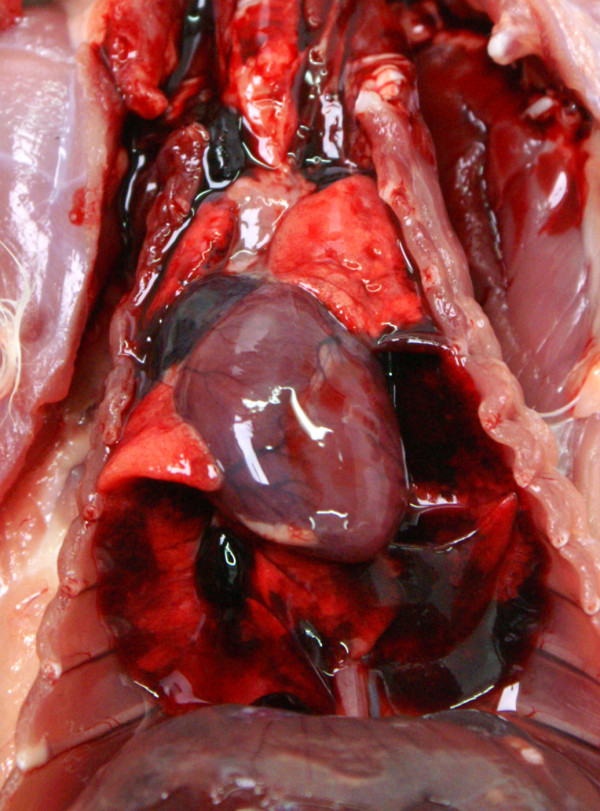
**Electrocution.** Extensive pulmonary haemorrhage in a squirrel that was electrocuted after climbing a mains electricity utility pole.

Extensive pulmonary haemorrhage was seen in an adult squirrel that died of electrocution in Scotland (Figure 12). It was found at the base of an electricity utility pole and had burn marks around the mouth and to the plantar surface of both hind feet and several large haemorrhages in the lungs. Apart from burns and pulmonary haemorrhage, no other lesions were seen on either gross or histological examination.

A squirrel found dead in a water butt was thought to have drowned. On histological examination of lung the alveoli appeared distended with acellular material containing various types of bacteria.

### Hepatobiliary disease

Lesions of varying severity were present in approximately 58.9% (56 of 95) of the livers that were suitable for histological interpretation. The most frequent were small, scattered, foci of hepatocyte necrosis associated with lymphocytes and mononuclear cells, often with a periportal distribution. The aetiology of these lesions was not apparent but they were considered to be of limited pathological significance. More extensive pathological changes were present in 40% of livers (38 of 95), with almost half of these due to infection with *Toxoplasma gondii* (n = 12) or *Capillaria hepatica* (n = 5).

**Figure 13:**
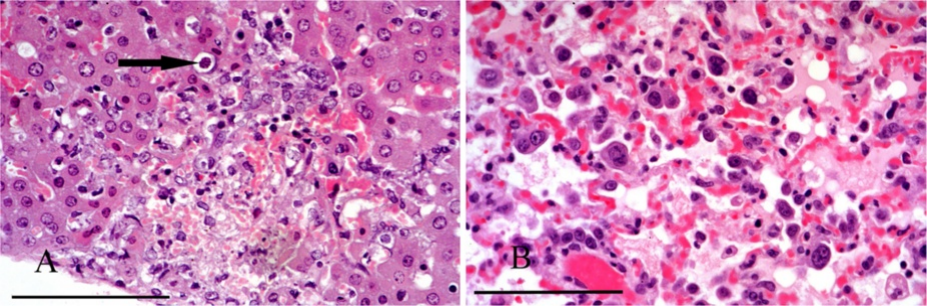
**Toxoplasmosis. A**: Focal hepatic necrosis and haemorrhage in a case of toxoplasmosis. A *Toxoplasma* cyst is present in the margin of the lesion (arrow). **B**: Pneumonitis caused by *T. gondii.* There is alveolar congestion and inflammatory cell infiltration with intraluminal and intramural macrophages and alveolar proteinaceous fluid. H & E Stain, bar = 100 mμ.

In cases of toxoplasmosis there was typically widespread multifocal, often periportal, hepatocyte necrosis with lymphocytic infiltration. *Toxoplasma* cysts were mostly present in hepatocytes around the margins of the lesions (Figure 13A). Affected squirrels often had similar lesions of multifocal necrosis in the spleen and in some cases pneumonitis (Figure 13B) and focal cardiac myopathy. All the cases of toxoplasmosis occurred in squirrels from the IoW and, of the 77 livers from there that were suitable for histological examination, 12 (15.6%) proved positive (Table 3). There was weak evidence for a difference in incidence of *T. gondii* between IoW and Scotland (p = 0.064). Nine of the 12 affected squirrels were females. Tissues from eight cases were examined by immunohistochemistry and in seven the parasite cysts and/or tachyzoites labelled positive for *T. gondii*. In the eighth case cysts seen in lung and liver lesions labelled negative despite having typical morphology.

**Figure 14:**
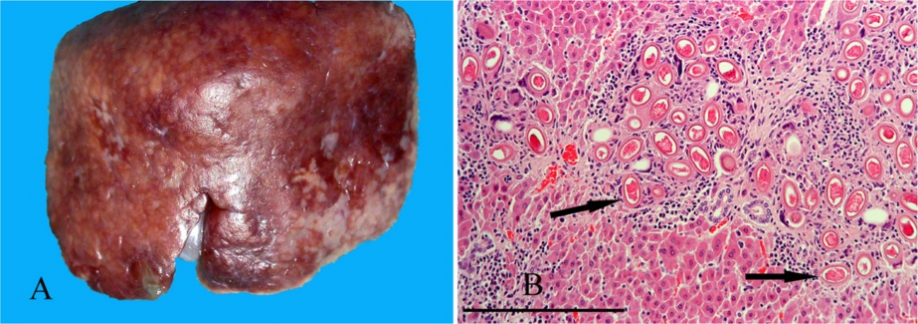
**Hepatic capillariasis. A**: Lobe of liver affected by capillariasis showing an irregular surface and pale mottled areas. **B**: Histological section of liver with clusters of *Capillaria* sp. eggs (arrows) enclosed in granulomatous reaction and fibrous tissue. H & E Stain, bar = 200 mμ.

Gross examination of livers affected by capillariasis showed lesions in three ranging from focal discolouration to swollen lobes with irregular or distorted surfaces (Figure 14A). Histologically all showed multiple focal clusters of eggs with bipolar plugs characteristic of *Capillaria* sp. and, in some cases, sections through adult nematodes. These were surrounded by granulomatous masses composed of macrophages, eosinophils, neutrophils and fibrous tissue which obliterated much of the parenchyma (Figure 14B). Four cases came from IoW and one from Scotland (Table 3).

Three livers showed lesions of multifocal coagulative necrosis associated with bacterial colonies, in one of which the lesions extended to involve the gall bladder. Unfortunately, liver was not made available for bacterial culture in any of these cases.

### Renal disease

**Figure 15:**
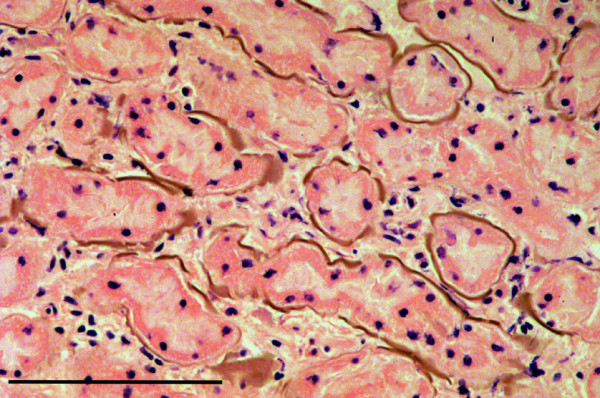
**Suspected cholecalciferol poisoning.** The brown staining of the renal proximal collecting tubules basement membranes is indicative of widespread deposition of calcium and is consistent with a diagnosis of cholecalciferol poisoning. Von Kossa stain, bar = 100 mμ.

Kidneys from 60 squirrels were suitable for histological examination and lesions were observed in 24 cases (40%). A squirrel that had been found dead on IoW had lesions suggestive of cholecalciferol rodenticide poisoning. There was extensive mineralisation of the proximal tubule basement membranes and mineralised castes in the medullary tubules but no significant inflammatory reaction; in sections stained by Von Kossa stain the mineralised deposits proved positive for calcium ions (Figure 15).

**Figure 16:**
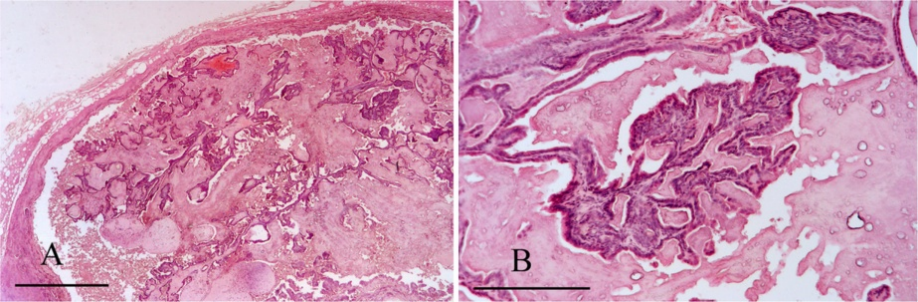
**Renal papillary adenoma. A**: Low magnification image of kidney showing proliferating cords of epithelial cells within a cystic structure that is compressing the adjacent parenchyma. The tissue is markedly autolysed. H & E Stain, bar = 1 mm. **B**: Higher magnification image of the same kidney section showing a proliferating, frond-like cord of the tumour. H & E Stain, bar =200 mμ.

A cystic renal papillary adenoma was seen in a squirrel that had been killed by road traffic. Although affected by autolysis, a large circumscribed cystic structure was clearly evident in the renal parenchyma. This had an irregular lining of epithelial cells and large, proliferative cords of papillary epithelium extending into the lumen (Figure 16).

Other renal lesions seen but thought to be of limited clinical significance included cystic collecting tubules (n = 2), lymphocytic interstitial nephritis (n = 6), small cortical infarcts with associated lymphocytic infiltrates (n = 4) and crystalline, possibly urate, deposits in medullary tubules (n = 4).

### Cardiovascular disease

The heart was examined histologically in 128 of 155 squirrels. Thirteen were unsuitable due to autolysis but lesions were seen in 27 of the remaining 115 cases. The great majority of these squirrels came from the IoW where approximately 21% (20/97) had multiple small foci of lymphocytic infiltration and in some cases, myocyte necrosis. In two such cases tachyzoites typical of *T. gondii* were observed in degenerating myocytes and toxoplasmosis was confirmed by examination of other organs. The aetiology of the lesions in the remaining 17 cases was not clear but on examination of other organs it was noted that toxoplasmosis was confirmed in four of the cases and suspected in a further five. In several squirrels small ovoid structures consistent with *Hepatozoon* sp. gamonts were observed lying between myofibres in myocardium.

### Splenic disease

During post-mortem examination spleens were observed to vary greatly in size but no significant gross pathological lesions were seen. Histologically, 35 of 45 spleens submitted were suitable for examination and lesions were seen in 11 (31.4%). In four cases the changes were of a minor nature, such as depletion of white pulp, but in seven there were multiple foci of necrosis, predominantly in the red pulp, associated with *Toxoplasma* cysts. Three of seven spleen samples screened by PCR for adenoviral DNA proved positive (Scotland 2, Anglesey 1) but none showed histopathological lesions.

### Alimentary tract disease

**Figure 17:**
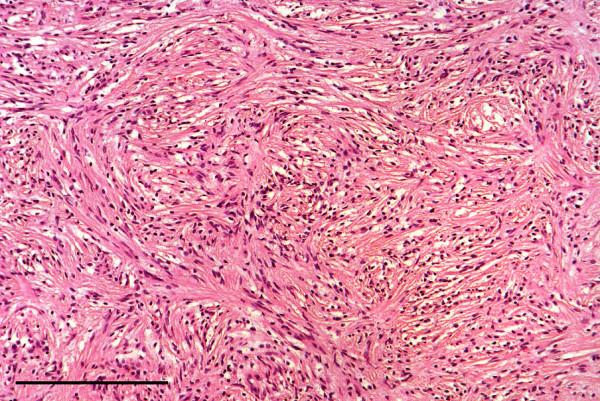
**Gastric spindle cell tumour.** The smooth muscle layer of the stomach wall is effaced by proliferating bundles of spindle cells. H & E Stain, bar = 200 mμ.

Oral lesions were a relatively important component of alimentary tract disease. Two squirrels with unevenly worn lower incisor teeth had unilateral osteomyelitis of a lower mandible associated with a facial abscess in one case and suspected bite wounds in the other. Penetration by a foreign body may have been responsible for a pharyngeal abscess in a squirrel that died of chylothorax. Lingual and laryngopharyngeal necrotising ulceration associated with *S. aureus* infection was considered to have predisposed to inhalation pneumonia in a juvenile squirrel on IoW and oropharyngeal/oesophageal candidiasis caused the death of a juvenile from Scotland. (For further detail see Simpson et al. [6]). Gastritis in a second juvenile from Scotland was associated histologically with a yeast infection but although the organism was isolated it could not be identified by API test. Coffee-ground haemorrhages and pyloric ulcers, thought to be stress-related, were seen in three cases. Post-mortem change often precluded detailed examination of the alimentary tract but 13 cases were examined histologically. An adult road traffic victim from the IoW had a large subserosal swelling of the stomach wall and several circumscribed swellings up to 7 mm diameter in the adjacent mesentery. The lesion in the stomach wall was a tumour composed of proliferating bundles of spindle cells that effaced the smooth muscle layer (Figure 17). The swellings in the mesentery were also composed of masses of spindle cells. The tumour distribution suggested multicentric origin. Histological features are often not predictive of malignant potential for gastro-intestinal spindle cell tumours and in the absence of immunohistochemistry further classification was not possible. The tumour(s) was therefore considered to be a spindle cell tumour of uncertain histiogenesis and biological behaviour.

Although signs of diarrhoea were occasionally observed during post-mortem examination of squirrels on the IoW, none of the 51 squirrels examined post-mortem at the Wildlife Veterinary Investigation Centre showed gross evidence of enteritis. Faecal samples from 33 squirrels (IoW 10, Scotland 20, Anglesey 3) were screened for adenovirus infection by TEM and all proved negative, despite spleen samples from three squirrels testing positive by PCR. Nematodes were sometimes observed during gross and histological examination of intestine. Morphologically they resembled pin worms (*Enterobius* sp.) but, apart from one case where they were identified as *Rodentoxyuris sciuri*, none were submitted for species identification. Typically there was no apparent associated pathology but one juvenile with a heavy nematode infection died due to an intestinal intussusception. However, in a second case of intussusception involving an adult there was no significant worm burden and the aetiology was obscure. Small to moderate numbers of a variety of *Eimeria* sp. coccidial oocysts were frequently observed in wet preparations of gut contents but without any associated pathology.

### Miscellaneous disease

A squirrel found dead on IoW had an enlarged, inflamed uterus and histological examination revealed the remains of a part-mummified foetus with trabecular bone evident. Unfortunately, it was too badly affected by autolysis for further examination. A second squirrel from IoW had extensive adhesions involving the abdominal organs and an unidentified mass in the peritoneal cavity. Histological examination of the mass showed it to be the necrotic remains of what was judged to be a foetus, possibly the result of an ectopic pregnancy.

## Discussion

Road traffic accident deaths were responsible for 41.7% of mortality overall and there was no significant difference between locations. This result is consistent that obtained LaRose et al. [9] in Scotland where 42.9% of mortality was caused by traffic. A slightly lower figure of 36% was recorded in 36 squirrels found dead on the island of Jersey [14]. The impact of road traffic mortality on red squirrel populations is uncertain. On the IoW at least, squirrel numbers appear to have been stable in recent years, although there was a suspected decrease during 2012 (Butler H., unpublished data). Trauma due to attack by predators, principally cats and dogs, was 9.2%. Concern has been expressed by Duff et al. [15] at the level of red squirrel predation by pets and on the island of Jersey 36% of squirrel mortality was attributed to attack by domestic cats [14]. This problem is in large part due to squirrels being attracted into gardens by people providing supplementary food. Although this may be beneficial from a nutritional point of view [14] it also increases the risk of predation.

Gross and histopathological examination identified pathological lesions associated with disease in 57 (35%) squirrels. The most important of these was toxoplasmosis. The susceptibility of red squirrels to this condition was first reported by Coles [16] in 1914 but in recent years it has become increasingly recognised as a cause of mortality [5,17,18]. In this study it was notable that all the toxoplasmosis cases were confined to the IoW where it was estimated to have caused almost one tenth of all deaths. The practice of providing supplementary food for squirrels is common on IoW and squirrels foraging in gardens are at increased risk of ingesting food contaminated by cat faeces. The human population density in the IoW is relatively high whereas at the other locations in the study, particularly in north east Scotland, the environment is much more rural with a low human, and presumably cat, population density. A second point of note is that three quarters of the toxoplasma-affected squirrels were females. This apparent sex bias does not appear to have been recorded previously and, whilst the reasons for it are not apparent, it could well be important as regards population recruitment.

In the study of red squirrels in Scotland [9] squirrelpox was the second most predominant cause of mortality (14.3%). However, squirrelpox does not occur on the IoW and was not seen in any of the squirrels submitted from other locations in this study. This was not unexpected in the case of squirrels submitted from Scotland as the majority came from an area believed to be free of squirrelpox; in addition, any Scottish red squirrels showing pox-like lesions are normally submitted direct to Edinburgh University for their pox screening program (http://www.red-squirrels.org.uk/surveillance.asp).

Thirty two squirrels were in poor or emaciated condition and half of these were juveniles or subadults. However, in no case was starvation diagnosed as the sole or primary cause of death. In some cases a heavy ectoparasite burden probably contributed to mortality but this was considered secondary. This result is in contrast to that obtained by LaRose et al. [9] in Scotland where starvation was the most common cause of mortality in juveniles and, at 9.8%, the fourth most common cause of death overall. A possible explanation for this is the additional laboratory procedures carried out in the present study. Anaemia associated with severe louse infestations has been reported as a likely cause of death in juvenile squirrels [9,15]. In this study, although heavy louse infestations were seen in six cases, in only one, a juvenile, was there obvious anaemia. It should be noted that whilst *N. sciuri* was the predominant louse species, some squirrels were also infested with *Enderleinellus nitzschi*. Both species have been recorded previously in red and grey squirrels in Great Britain [19] but in most publications it would appear that the identification of *N. sciuri* is presumptive [8,9,11,20]. *Neohaematopinus sciuri* has a Holarctic distribution and *Enderleinellus* species are found worldwide [21]; it is therefore surprising that in this study lice were only found on squirrels from Scotland. The apparent absence of lice on squirrels submitted from Cumbria and Anglesey may be because so few were examined from these locations but this does not explain the apparent absence of sucking lice from the IoW. The authors accept that light burdens might have been missed during these post-mortem examinations but it is highly unlikely that this would have happened with heavy infestations. Similarly, ticks were only observed in squirrels from Scotland although they have been observed historically on IoW (H. Butler, unpublished data). In view of the apparent absence of both parasites from squirrels on IoW it is of interest that lice were not recorded, and ticks rarely so, in a post-mortem study on red squirrels from the Island of Jersey [T. Blackett, personal communication].

*Capillaria hepatica* is capable of infecting a wide range of species but is primarily a parasite of rodents. The life cycle is dependent on the death of the host and digestion of the liver to release the eggs. Typically this occurs through predation, cannibalism or necrophagy. A new host only becomes infected by ingesting embryonated eggs that have been excreted in the faeces of the predator or scavenger. As the highest prevalence of the parasite is normally in commensal populations of brown rats (*Rattus norvegicus*) [22] it is likely that rats are the main source of infection in squirrels. Rat infestations are a common problem in gardens where people feed squirrels and birds. In this study it was difficult to assess the pathological significance of the lesions caused by *C. hepatica*. Four of the five infected squirrels were in good condition and were killed by road traffic whilst the fifth was suffering from a severe facial abscess and mandibular osteomyelitis. However, in other studies [17, Simpson, V. unpublished data] mortality has been associated with severe parasite-induced liver lesions in both wild and captive red squirrels.

Apart from *T. gondii*, the importance of protozoal infections in red squirrels is uncertain. Infection with *Hepatozoon* sp. was common, especially on the IoW where a third of all squirrels were infected. Heavy *Hepatozoon* burdens were most commonly seen in squirrels suffering other concurrent respiratory infections but it is unclear whether a high *Hepatozoon* burden predisposes squirrels to these infections or whether high burdens are a consequence of other infections or stress factors. There was no clear evidence that the parasite on its own was causing mortality. A similar situation possibly exists with coccidiosis. Keymer [8] suggested that this is a common cause of death in red squirrels but in the present study it was apparent that, whilst *Eimeria* spp infection was common, there was no evidence that this was associated with disease. Sainsbury and Gurnell [23] came to the same conclusion in a study of red squirrels in Norfolk and Sainsbury [20] suggests that, as in other mammalian species, disease in red squirrels due to coccidia is precipitated by stressors or concomitant disease. Severe coccidiosis has been described in squirrels dying of colonic intussusception [11] but there was no evidence of coccidiosis in the two cases of intussusception in the present study.

An enteric adenovirus was first reported as a suspected cause of enteritis and mortality in red squirrels translocated from Cumbria to Norfolk [3]. Since then there is increasing evidence that the virus is associated with fatal enteritis in wild and, particularly, in captive red squirrels in England, Wales, Scotland and Northern Ireland [4,11,24,25]. However, although PCR analysis on spleens demonstrated adenoviral DNA in three squirrels in this study, and in four of seven squirrels from IoW examined as part of another study (Everest and Butler, unpublished data), in no case was there evidence of associated pathology. This suggests that in many cases adenovirus infection in wild red squirrels is asymptomatic.

Keymer [8] suggested that bacterial infections in red squirrels are rare but in this study they were quite frequent, particularly those affecting the respiratory system where histological examination showed lesions associated with bacteria in approximately 11% of cases. However, in many cases the infection appeared to secondary, for example in squirrels dying of inhalation pneumonia. In the study by LaRose et al. [9] of red squirrel mortality in Scotland 7.3% of the deaths were attributed to pneumonia but as this diagnosis was based on gross pathology only it is not possible to make a direct comparison with the present study.

The most important bacterial infection in this study was *Staphylococcus aureus* associated with fatal exudative dermatitis in IoW squirrels. This condition is also a major cause of red squirrel mortality on the island of Jersey [7] and the *S. aureus* isolates from affected squirrels on both islands are of the same lineage and all encode the *luk*M gene [13]. The pathogenesis of the condition is not understood but it is invariably fatal. Further studies into factors that may predispose squirrels to *Staphylococcus*-associated fatal exudative dermatitis are in progress.

*Bordetella bronchiseptica* is a common cause of respiratory disease in dogs and cats but is only occasionally recorded in wildlife. In most cases bordetellosis is seen as a secondary or opportunistic infection in stressed or compromised animals, for example dogs and cats in boarding kennels or seals affected with phocine distemper [26-28]. In this study, acute, fatal bronchopneumonia due to *B. bronchiseptica* was diagnosed in two squirrels and was suspected in a third. There was no evidence to suggest that these were secondary infections although they did have heavy *Hepatozoon* infections. The source of infection was not apparent.

Neoplastic disease is uncommon in most British free-living wild species but red squirrels appear to be unusually susceptible. Cases of soft tissue sarcoma and lymphoma have been recorded in squirrels in Scotland [9], two cases of lymphosarcoma in Wales [11] and pulmonary adenomatosis and lymphoma in Jersey [29]. Neoplasms seen this study were pulmonary carcinoma, trichoepithelioma, gastric spindle cell tumour and renal papillary adenoma. In addition, four squirrels, three from IoW and one from Brownsea, had unusual lesions of epidermal hyperplasia of the ear pinnae associated in two cases with cutaneous wart-like growths. None of the neoplastic lesions observed in this study appear to have been recorded previously in red squirrels and it was notable that all occurred in cases from IoW. A preliminary investigation in to the possibility that red squirrels, in common with other *Rodentia*[30] carry Gammaretroviruses that are capable of acting as etiological agents for transmissible neoplasia indicated the presence of one or more potentially infectious retroviruses [Tarlinton and Lucassen personal communication].

## Conclusion

As the grey squirrel population continues to extend its range over Great Britain, the viability of the increasingly isolated red squirrel populations becomes doubtful. Even in areas where squirrelpox does not currently occur red squirrels suffer premature mortality from additional causes, many of which are due, directly or indirectly, to human activities. Road traffic trauma, pet predation, toxoplasmosis, trap injuries, rodenticide poisoning and electrocution accounted for 61% of all recorded mortality in this study. There is also evidence that infectious agents such as adenovirus may be spread during translocation or reintroduction programmes [3,11]. The impact of human activities on the health and welfare of red squirrels in Great Britain merits more attention than it has received in the past. There is also a need for continued disease surveillance. As this study has shown, red squirrels are affected by a wider range of diseases than previously described.

## Competing interests

The authors declare they have no competing interests.

## Authors’ contributions

VS conceived, designed and coordinated the study, participated in the gross and histopathology and drafted the manuscript, HB participated in the design of the study and the gross pathology, JH participated in the histopathology, ND performed the bacteriology and DE performed the virology. All authors contributed to writing the draft manuscript and all read and approved the final manuscript.
